# Bacteria associated with moon jellyfish during bloom and post-bloom periods in the Gulf of Trieste (northern Adriatic)

**DOI:** 10.1371/journal.pone.0198056

**Published:** 2019-01-15

**Authors:** Maja Kos Kramar, Tinkara Tinta, Davor Lučić, Alenka Malej, Valentina Turk

**Affiliations:** 1 Marine Biology Station Piran, National Institute of Biology, Piran, Slovenia; 2 Department of Limnology and Bio-Oceanography, Center of Functional Ecology, University of Vienna, Vienna, Austria; 3 Institute for Marine and Coastal Research, University of Dubrovnik, Dubrovnik, Croatia; University of Illinois at Urbana-Champaign, UNITED STATES

## Abstract

Jellyfish are a prominent component of the plankton community. They frequently form conspicuous blooms which may interfere with different human enterprises. Among the aspects that remain understudied are jellyfish associations with microorganisms having potentially important implications for organic matter cycling. To the best of our knowledge, this study is the first to investigate the bacterial community associated with live moon jellyfish (*Aurelia solida*, *Scyohozoa*) in the Adriatic Sea. Using 16S rRNA clone libraries and culture-based methods, we have analyzed the bacterial community composition of different body parts: the exumbrella surface, oral arms, and gastric cavity, and investigated possible differences in medusa-associated bacterial community structure at the time of the jellyfish population peak, and during the senescent phase at the end of bloom. Microbiota associated with moon jellyfish was different from ambient seawater bacterial assemblage and varied between different body parts. *Betaproteobacteria* (*Burkholderia*, *Cupriavidus* and *Achromobacter*) dominated community in the gastral cavity of medusa, while *Alphaproteobacteria* (*Phaeobacter*, *Ruegeria*) and *Gammaproteobacteria* (*Stenotrophomonas*, *Alteromonas*, *Pseudoalteromonas* and *Vibrio*) prevailed on ‘outer’ body parts. Bacterial community structure changed during senescent phase, at the end of the jellyfish bloom, showing an increased abundance of *Gammaproteobacteria*, exclusively *Vibrio*. The results of cultured bacterial isolates showed the dominance of *Gammaproeteobacteria*, especially *Vibrio* and *Pseudoalteromonas* in all body parts. Our results suggest that jellyfish associated bacterial community might have an important role for the host, and that anthropogenic pollution in the Gulf of Trieste might affect their community structure.

## Introduction

Jellyfish are important consumers of diverse plankton prey, from protists to small fish [1–3], and have value as prey for a range of different animals [4,5] including other jellyfish to large fish, turtles, and birds [6,7]. It was estimated [8] that the global median gelatinous plankton biomass of the epipelagic ocean was 0.81 mg C m^-3^, to which a majority (> 90%) was contributed by jellyfish (*Cnidaria*, *Ctenophora*). Global jellyfish outbreaks seem to have become more frequent and last longer in recent years [9]. Whether this is just a rising phase of a natural pattern of decadal oscillations, or a true increase of gelatinous zooplankton blooms is still unclear [10]. Still, some data show more frequent and abundant jellyfish aggregations in variable coastal areas around the world [11,12]. It has been hypothesized that jellyfish have benefitted from human-caused changes in environment, such as: climate change, overfishing, eutrophication, habitat modification, and species introductions [13–16].

The jellyfish outbreaks have provoked concern about their potential harm for human well-being [17] and stimulated research on jellyfish in the past two decades. Through the process of carbon sequestration, jellyfish provide regulating services [18], contribute nutrients to support primary production [19] and organic matter that stimulates microbes. Several studies have investigated the role of bacteria during jellyfish blooms. High bacterial growth, changes in the bacterial community structure in the surroundings of live or decaying jellyfish, and subsequent consequences in altering trophic interactions with higher trophic levels have been demonstrated together with implications for the carbon, nitrogen, and phosphorus cycles [20–29].

The surfaces of marine animals were found to be a unique habitat for colonization by microorganisms, and the microbial communities associated with living surfaces showed a pronounced variety [30]. Till recently, studies have focused on the colonization of benthic organisms such as sponges [31–35], bryozoans [36], and cnidarians, within which are included mainly corals [37–42]. Current studies of bacteria colonizing crustacean surfaces in the marine pelagic environment have shown considerable dissimilarities with bacterial communities in the surrounding seawater [43–45]. Recently, associated bacteria were reported for gelatinous plankton such as ctenophores [46–49]. Studies on cnidarian jellyfish show the presence of endobiotic bacteria in jellyfish tentacles [50], and suggest that jellyfish could be vectors of bacterial pathogens and implicated in infections of farmed salmons [51,52]. Cleary *et al*. [53] presented data on the bacterial community composition associated with scyphozoan *Mastigias* cf. *papua etpisoni* and box jellyfish *Tripedalia cf*. *cystophora*, while Weiland-Bräuer *et al*. [54] and Daley *et al*. [46] focused on *Aurelia aurita* s.l. bacterial associates. These studies showed a diverse and specific bacterial community associated with jellyfish, which differs in composition among different marine ecosystems/ different jellyfish populations, and has little similarity to the surrounding seawater. Furthermore, Weiland-Bräuer *et al*. [54] showed that *A*. *aurita* harbors a different bacterial community on its outer, mucus-covered surface of the exumbrella and gastral cavity, and that microbial community composition differs at different life stages, especially between benthic (polyps and strobila) and sequential planktonic life stages (ephira and juvenile and adult medusa). Studying microbiomes in the gastral cavity of *Cotylorhiza tuberculata*, *Mycoplasma*-like bacteria was one of four bacterial taxa composing a community of reduced diversity [55,56]. Some of the bacteria were suggested to have an intracellular lifestyle, established a cooperative relationship with their host [55].

Bacterial colonization of a given surface is determined by the availability of nutrients, host immune responses, and competition between bacteria from the surrounding environment for attachment space [57]. The epidermis and gastrodermis of jellyfish, including *A*. *aurita*, contain numerous types of unicellular mucus producing gland cells, leading to the formation of thin, constantly renewing mucus layers over external surface of medusa [58,59]. Under certain conditions like stress, during reproduction and digestion, and also when dying, the amount of released mucus is even more pronounced [59]. Mucus on jellyfish surfaces was also found to have a role in surface cleaning and defense against predators [59]. Shanks and Graham [60] characterized mucus secretion as an important chemical defense mechanism since it contained toxins and discharged and undischarged nematocysts. The contribution to jellyfish chemical defense is, besides mucus, the production of toxins or antimicrobial compounds, such as isolated antibacterial peptide aurelin from mesoglea of *A*. *aurita* [61].

Further, secreted mucus is an attractive niche for bacteria. Since jellyfish mucus is composed mainly of proteins, lipids, and a lower percentage of carbohydrates [62], it is a high quality energy source which is readily utilized by bacteria, especially those with a competitive advantage and specialized for settling from surrounding seawater. This indicates that jellyfish as a host can actively or passively affect/select bacterial associates. In addition, bacterial community structure can be also influenced by a bacterium-bacterium antagonism, as seen on particles [63], and by environmental conditions determining the presence of metabolically active bacteria and physiological responses of the host [64]. Whether bacteria directly adhere to external cell layers of jellyfish or are only associated in the thin mucus layer is not clear, however, all the above indicates that the association of bacteria with jellyfish is highly dynamic and complex.

This study is the first to investigate the associations of bacteria with live moon jellyfish using both culture-independent and culture-based methods, and it is also the first to be carried out in the northern Adriatic Sea, where moon jelly, *Aurelia* sp. 8 [65], recently designated as *A*. *solida* [66], is a very common jellyfish, and where 200 years of data show the stabilization of its massive reoccurrence after 2002 [12].

Our hypotheses were the following: (i) the bacterial community associated with medusa is specific and different from the ambient bacterial population in the environment; (ii) the bacterial community composition of different body parts of medusa, i.e. the exumbrella surface, oral arms, and of gastral cavity vary; and (iii) medusa-associated bacterial community structure at the time of jellyfish population peak and during senescent phase at the end of bloom, when jellyfish start to decay, differ.

## Materials and methods

### Sampling and sample preparation

The Gulf of Trieste is the northernmost part of the Adriatic Sea. It is characterized by a shallow water column, with salinity and temperature variations, and strong seasonal stratification in late summer [67]. In such an environment, *Aurelia* populations show clear seasonality with late autumn/early winter recruitment of ephyrae from attached polyps, spring medusa growth, and their decay at high early summer temperatures [16]. Seasonal blooms of *Aurelia* sp., when medusae reach very high abundance, depend on benthic polyp production of young medusae (strobilation period), which occurs in the northern Adriatic from November to March [68]. Maximal medusae densities occur from February to May and decline in June with increasing sea’s temperatures [69].

Sampling of *Aurelia* sp.8 [65], recently designated as *A*. *solida* [66], was performed in the beginning of May and late June 2011. While in May, at the time of population peak, jellyfish were viable and swimming actively, in June at the end of the blooming period, sampled jellyfish were already in the senescent phase and started to decay. Senescent medusae showed reduced activity (as assessed by bell pulsation rates), changes in bell consistency, and increased proportion of damaged individuals. Sampled individuals did not contain planulae while sex was not determined. Jellyfish were sampled individually by divers, or from a boat with a sample bucket. Each individual sample was placed in a plastic bag with some seawater and was transported to the laboratory. Before further analysis, each jellyfish was measured and rinsed twice with sterile seawater (0.2 μm pre-filtered and autoclaved). Each time 20 jellyfish were collected (10 for total community analysis and 10 for culture-based analysis) with the bell diameter ranging from 10 to 17 cm in May, and from 12 to 21 cm in July. For determination of the total bacterial community associated with *Aurelia*, samples of exumbrella and oral arms of about 8 cm^2^ in size, were cut out with a sterile razor blade and stored at -80°C. At the same time, mucus from gastral cavity was sampled with a sterile syringe and stored under the same conditions. At the same time of the medusa sampling, ambient seawater samples were collected with a Niskin sampler (V = 5 L) at 5 m depth at the oceanographic buoy Vida (45° 32’ 55. 68” N, 13°33’ 1.89” E), where most of jellyfish were restrained at the time of sampling. Seawater samples were transferred to the laboratory, where they were immediately filtered onto 0.2 μm polyethersulfone sterile membrane filters (47 mm diameter, PALL Inc.), and stored at -80°C. Each time before sampling, standard physical properties including seawater temperature, salinity, and oxygen concentration were measured with a CTD fine-scale probe (Microstructure Profiler MSS90, Sea & Sun Technology GmbH).

### Total bacterial community composition

#### Jellyfish-associated bacterial community DNA extraction

Exumbrella (four samples per month), oral arms (four samples per month), and gastral cavity (two samples in May) samples were thawed down and homogenized, from which 5 mL was used for bacterial DNA extraction. The DNA of the total bacterial community associated with *Aurelia*, was extracted with CTAB (cetyl-trimethyl-ammonium bromide) as described by Hao [49] with slight modification (see S1B Protocol)).

#### Seawater’s total bacterial community DNA extraction

DNA was extracted from the filters (one quarter per sample) according to Böstrom *et al*. [70], with slight modifications, as described before [25].

#### Bacterial 16S rRNA gene clone libraries

For jellyfish and seawater samples clone libraries construction, bacterial 16S rRNA gens were amplified using the universal primer set, 27F and 1492R (S2D Protocol; Standard clone library approach). For samples with low DNA concentration (extracted from jellyfish samples), a modified nested PCR-libraries approach was used [71]. Bacterial 16S rRNA gene was first amplified with a universal primer set, 27F and 1492R. Second, nested amplification was performed using primers 341F and 907R (S2D Protocol; Nested- PCR clone library approach). The PCR products were immediately ligated into a commercially available pCR 2.1 vector and transformed into competent *E*. *coli* TOP 10 cells using a commercially available TA Cloning Kit (Invitrogen), according to the manufacturer’s protocol. The plasmid inserts of each clone library were sequenced using M13F primer, 341F primer or 27F primer at Macrogen Inc.

#### Denaturing gradient gel electrophoresis

For the DGGE analysis the bacterial 16S rRNA genes were amplified using a universal primer set, 341F with a 40 bp GC-clamp and 907R [72,73]. The PCR touchdown protocol according to Don *et al*. [74] was used (see S2B Protocol; Standard PCR- DGGE strategy). To obtain a sufficient quantity of PCR products from jellyfish samples, we used a two-step nested PCR-DGGE strategy [75], with modifications. Bacterial 16S rRNA genes were first amplified with the universal primer set, 27F and 1492R and then, nested amplification was performed using a DGGE primer set, and a touchdown annealing protocol (S2B Protocol; Nested PCR- DGGE strategy). The quality and size of PCR products were confirmed by agarose gel electrophoresis. PCR products were analyzed by DGGE electrophoresis, as previously described in [25].

Distinct bands were excised from the gel and the eluted DNA was re-amplified using primer set 341F and 907R (S2C Protocol). The bacterial 16S rRNA genes were sequenced with 341F primer at Macrogen Inc.

### Bacterial isolates from jellyfish and seawater samples

#### Preparation of bacterial colonies and isolates

Viable bacterial cells from the surfaces of jellyfish and seawater samples were determined with the spread plate method on modified ZoBell marine agar [76]. The whole exumbrella surface was inoculated on the plate to create jellyfish imprints of exumbrella-associated bacteria, while the gastro vascular cavity was scraped with a sterile cotton swab and spread evenly over the surface of agar plates (with five jellyfish individuals for each sample type). For seawater samples, 100 μL was spread on an agar plates and inoculated plates were incubated in the dark at 17.0 ^o^C in May and 24 ^o^C in June, for 21 days. The number of colony-forming units (CFU) was determined, and distinctive morphological types of colonies were described for each plate.

For DNA extraction, all individual colonies were aseptically picked and streaked onto a fresh agar plate until single colonies were obtained. A single colony of each bacterial isolate was inoculated in modified liquid ZoBell media and incubated in the dark at 17.0 ^o^C in May and 24 ^o^C in June, until growth was observed (increased turbidity). Altogether, 135 bacterial isolates acquired from the exumbrella surface and gastral cavity of jellyfish sampled in May (AK1, AK3, AK6, AG1, AG6), and in June (AK8, AK10, AK11, AG8, AG11) were further used for DNA extraction.

#### Bacterial isolates' DNA extraction and PCR reaction

Bacterial cells were harvested from a liquid culture by centrifugation and washed twice with 1x PBS buffer. Bacterial DNA was extracted with a modified Chelex-based procedure [77] (S1A Protocol), or with a commercial kit (NucleoSpin Tissue, Macherey—Nagel) according to the manufacturer’s protocol. Bacterial 16S rRNA genes were amplified using universal bacterial primers 27F and 1492R [78]. The PCR reaction mix composition and PCR temperature cycling conditions are presented in the Supporting Information (S2A Protocol). The bacterial 16S rRNA genes were sequenced with 27F primer at Macrogen Inc.

### Sequence analyses

Raw sequence data recovered from sequencing 16S rDNA genes of bacterial isolates and bacterial 16S rDNA gene clone libraries were passed through the DNA Baser program (www.DNAbaser.com) to remove traces of sequencing primers, and to trim away ambiguous bases at the end of a sequence. The clone libraries sequences were also screened for vector contamination and analyzed with the program Bellerophon (https://greengenes.lbl.gov/) to detect chimeric sequences, which were removed from the batch. Additionally, Mothur software [79] was used to further reduce/remove poor quality sequence data and to assign sequence taxonomic identities of bacterial isolates and sequences recovered from clone libraries and DGGE bands, according to SILVA reference database (release 102) by using Wang approach with 80% bootstrap value. Taxonomic classification of sequences recovered from clone libraries and DGGE bands was done down to the bacterial family level. Only the batch of sequences that we were able to classify down to the family level was subjected to further statistical analyses. The rest were taken into account only when describing general diversity differences.

Taxonomic classification of bacterial isolates was done down to the genus level. The number (N) of high quality sequences obtained from clone libraries and by culturing is presented in Supporting Information (S1 Table, S2 Table). The contribution of distinct bacterial taxa was expressed as a percentage of the total number of sequences in each sample or library (relative abundance) (S1 Table, S2 Table). Chloroplast sequences were omitted from further analysis.

### Nucleotide sequence accession numbers

The 16S rRNA gene sequences, for all bacterial isolates, clone libraries, and DGGE bands obtained in this study were deposited in the GenBank (NCBI) under following accession numbers: from KF816449 to KF816471, and KF816480 to KF816592 for bacterial isolates (Supporting information, S5 Table), from KF816761 to KF816832, from KF817469 to KF817519, from MF952738 to MF952748, and from MF952764 to MF952865 for sequences obtained from clone libraries, and from MF952749 to MF952763 for sequences obtained from DGGE bands.

Data on the seawater cultural bacterial community from the Gulf of Trieste were compared with the dataset gathered during two-year sampling campaign of cultural bacterial community, of which the sampling time and location coincided with the sampling time of jellyfish (May and June 2011) (Acc. No. KC307273- KC307520).

For statistical analysis, additional data of the total seawater bacterial community collected from 5m depth at the oceanographic buoy Vida in May 2010 in the Gulf of Trieste was used (Acc. No. JX864324- JX864369; in [79]).

### Diversity indices and statistical analyses

To compare the diversity of bacterial community associated with jellyfish and the surrounding seawater, ecological diversity indices were calculated for each sample: the number of different bacterial genus (species richness (S)), Shannon diversity index (H’), Margalef’s index (d), Pielou’s evenness index (J’), and the Chao-1 index. Additionally, in order to estimate how well the actual species composition was captured, for each clone library a coverage value was calculated as C = 1-n_1_/N, where n1 is the number of phylotypes appearing only once in the library, and N is the library size [80].

Non-metric multi-dimensional scaling (nMDS) plots were used to determine the similarities between DGGE banding patterns. For this purpose, a similarity matrix was calculated (using Jaccard resemblance measure) based on the presence/absence matrix of align bands. Analysis of similarity (ANOSIM) was used to verify the significance of similarity among bacterial communities, as indicated by nMDS, by testing the hypothesis that bacterial communities from the same cluster are more similar to each other than to communities in different clusters.

Cluster analysis was used to determine scaled similarities between 16S rRNA gene clone libraries (total bacterial communities) and between bacterial isolates (culturable bacterial communities). For cluster analysis of 16S rRNA gene clone libraries, a Bray-Curtis similarity matrix was constructed from arcsine-transformed relative abundances of distinct bacterial families in each clone library. For bacterial isolates, a Bray-Curtis similarity matrix was constructed from untransformed relative abundances of distinct bacterial genus in each culturable bacterial community. Based on the similarity matrix, a dendrogram was produced with group-average linkage algorithm. The similarity profile test (SIMPROF) was used to define statistically significant clusters in samples.

To examine the difference between communities associated to different jellyfish body parts and seawater, one-way ANOSIM statistic with 999 permutations was made, based on Bray-Curtis similarity matrix. Samples were grouped according to isolation source (communities of jellyfish exumbrella (AK), jellyfish oral arms (AR) and jellyfish gastral cavity (AG), and communities of seawater (W)). Similarly, one-way ANOSIM statistic with 999 permutations was made to examine the difference between communities associated with jellyfish at the time of population peak and at the end of the blooming period. Additionally, similarities percentage (SIMPER) analysis was used to determine which bacterial groups contribute the most to the differences between communities. Diversity indices and statistical analysis were performed using Primer v6 [81] and PAST, version 3.9 [82].

## Results and discussion

### *Aurelia* associated *versus* ambient seawater bacterial community composition

**Fig 1:**
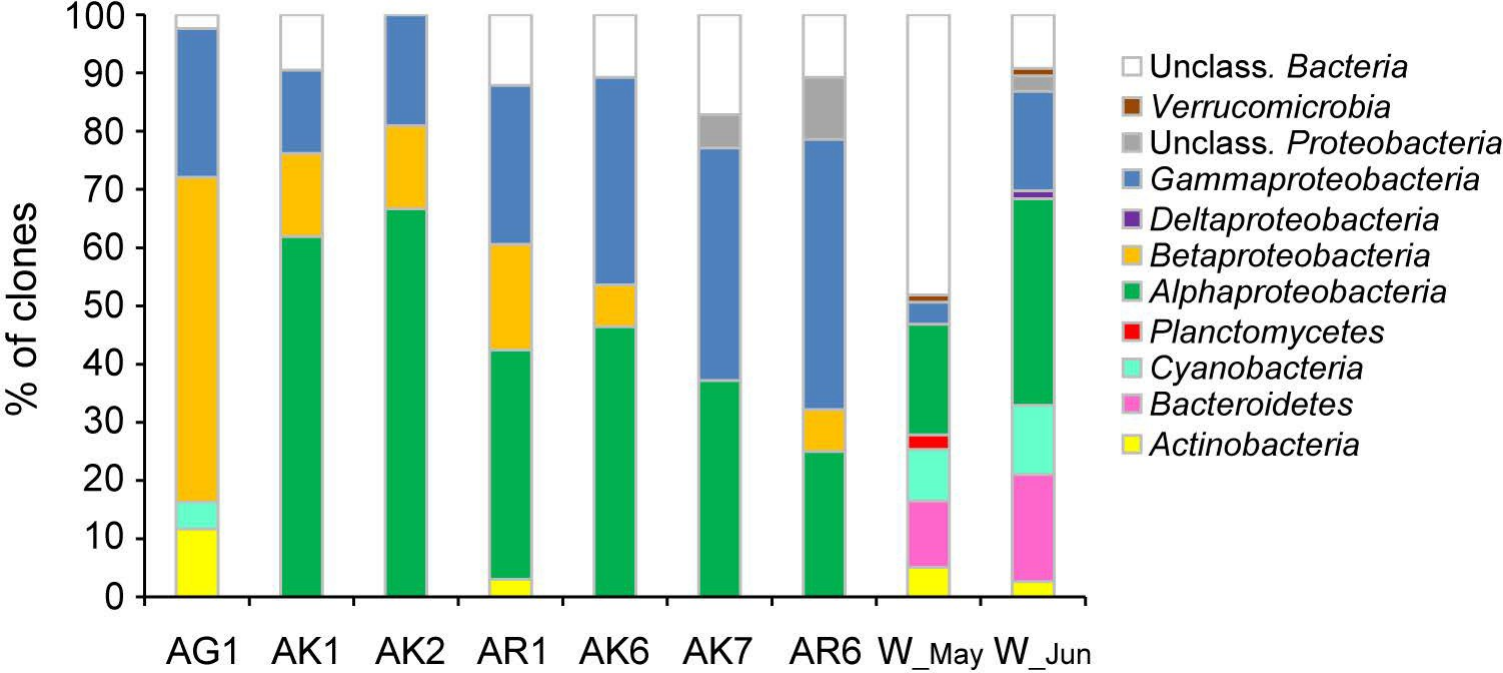
Bacterial 16S rRNA gene clone libraries constructed from samples of *Aurelia* jellyfish and ambient seawater. Cumulative bar charts comparing the relative abundances (% of clones) of main phyla and *Proteobacteria* class for samples of jellyfish exumbrella surface (AK1, AK2), oral arms (AR1), mucus from gastral cavity (AG1), ambient seawater (W_May) sampled in May and jellyfish exumbrella surface (AK6, AK7), oral arms (AR6), and the ambient seawater (W_Jun) sampled in June.

Phylogenetic analysis of 16 S rRNA clone libraries showed a diversity of bacterial community associated with jellyfish, including members of *Proteobacteria*, which dominated the community, and members of *Actinobacteria* and *Cyanobacteria* (Fig 1). Ambient seawater bacterial communities were more diverse, but dominated by *Proteobacteria*, *Flavobacteria*, and *Cyanobacteria* (Fig 1, S5 Table).

**Fig 2:**
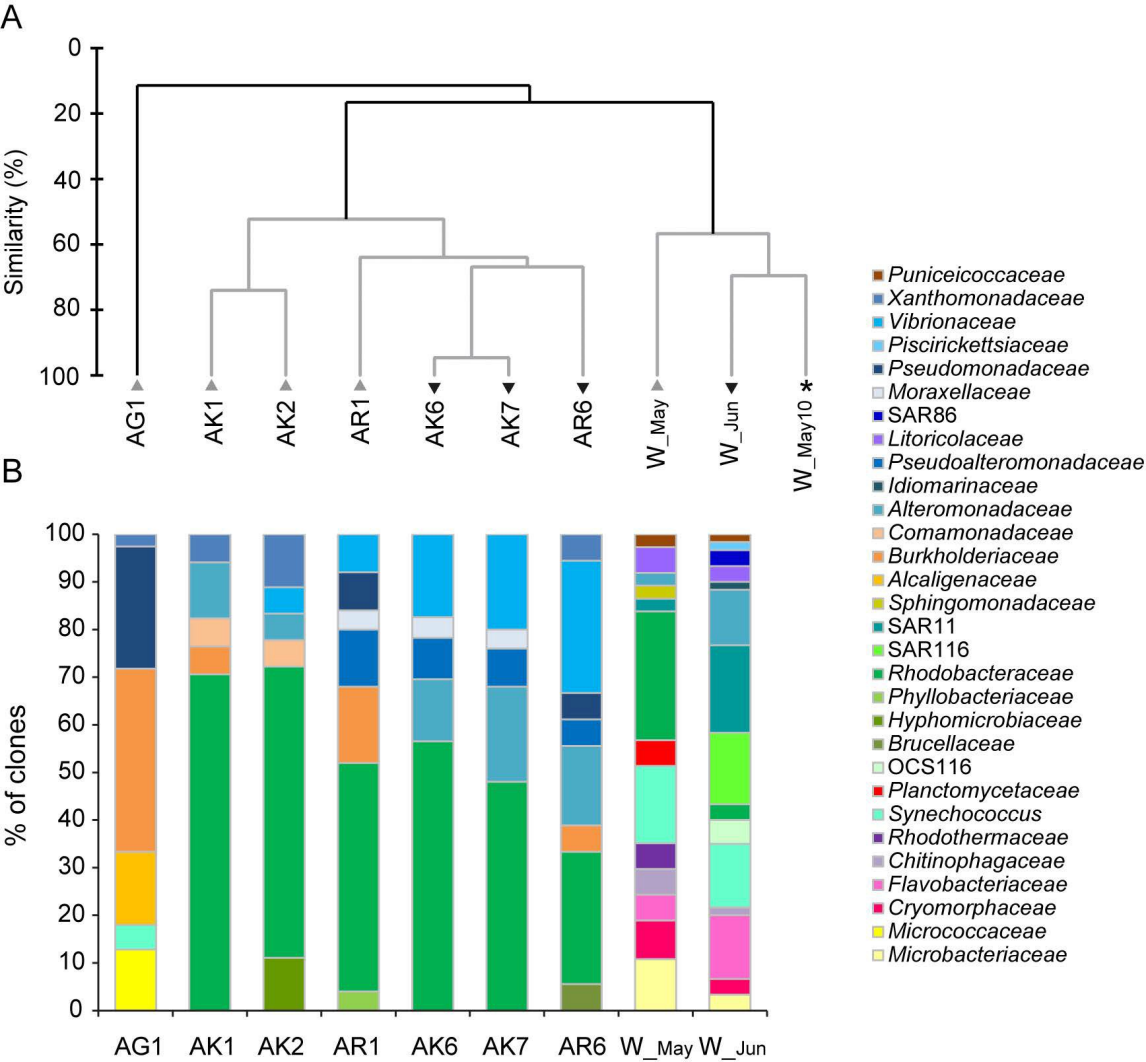
Bacterial community composition at the bacterial family level. (**A**) Cluster analysis based on bacterial community composition at the family level. AK-jellyfish exumbrella surface, AR-jellyfish oral arms, AG-mucus from gastral cavity and W-ambient seawater. Samples were collected in May (grey triangles) and June (inverted black triangles). Asterisk mark the additional water sample collected in May 2010 in the Gulf of Trieste. The dendrogram was inferred with the group average algorithm, based on the Bray–Curtis similarity matrix of arcsine transformed averaged abundances. The grey branches do not differ significantly (SIMPROF test, p> 0.05). (**B**) The dynamic of bacterial families within bacterial phyla and *Proteobacteria* class. Cumulative column charts represent relative abundances of bacterial families. Jellyfish exumbrella surface samples (AK1, AK2), oral arms sample (AR1), mucus sample from gastral cavity (AG1), ambient seawater (W_May) collected in May and jellyfish exumbrella surface samples (AK6, AK7), oral arms sample (AR6), and the ambient seawater sample (W_Jun) collected in June.

Our results on bacterial community composition at the family level, demonstrated differences between the bacterial community associated with *Aurelia* and the ambient seawater bacterial assemblage (ANOSIM, global R = 0.71, p< 0.05) (Fig 2A). Jellyfish associated bacterial community were within *Alphaproteobacteria* dominated by *Rhodobacteraceae* (mostly *Phaeobacter*, *Ruegeria*) and within *Betaproteobacteria* by *Burkholderiaceae* (*Burkholderia*). Within *Gammaproteobacteria*, mostly *Vibrionaceae* (*Vibrio*), *Pseudoalteromonadaceae* (*Pseudoalteromonas*), *Xanthomonadaceae* (*Stenotrophomonas*), and *Pseudomonadaceae* (*Pseudomonas*) (Fig 2B, S1 Table) were detected.

Ambient seawater communities were within *Alphaproteobacteria* dominated by *Rhodobacteraceae* and SAR11, within *Gammaproteobacteria* by *Litoricolaceae* and SAR86; within *Flavobacteria* by *Flavobacteriaceae* and *Cryomorphaceae*, and by *Synechococcus* (*Cyanobacteria*). We also detected *Actinobacteria* with the representative from the *Microbacteriaceae* family (Fig 2B, S1 Table). According to SIMPER analysis *Flavobacteriaceae*, *Synechococcus* and SAR11 which were characteristic for seawater assemblages, mainly contributed to difference between jellyfish-associated and water column bacterial community (S3 Table).

Similar observations of the jellyfish-specific bacterial community, distinct from the community in ambient seawater, were reported previously for *A*. *aurita* [46,54], and also other marine animals [64]. Since associated bacterial assemblages differed from the ambient seawater bacterial community, and from bacteria associated with other types of substrates/surfaces found in the water column, it was suggested that associations with animals might be specific to some degree [64]. According to Taylor *et al*. [83] sponge bacterial associates could be separated/split into three groups: (i) bacterial specialists–found on only one host species; (ii) host associates–found on multiple hosts; and (iii) generalists–found on multiple hosts and within the seawater community. In our study, most bacteria associated with *Aurelia* were not detected in the ambient seawater; however, they were closely related to bacteria previously found in association with other host animals, indicating that this relationship is not host-specific. Previous studies on *A*. *aurita* bacterial associates also did not reveal the presence of any *Aurelia*-bacterial specialists, with the exception of *Mycoplasma* sp. (class *Mollicutes*), a possible/hypothetical endosymbiont [46,54]. However, in our study, we were not able to detect any *Mycoplasma* members and the bacterial community composition was different to the community associated with *A*. *aurita* from the North West Atlantic and the Baltic Sea [46,54]. This might suggest the possible effect of host genetics background (different populations of *Aurelia* species in geographically distant locations), and the importance of environmental and anthropogenic conditions, determining the presence, activity, and composition of bacterial community in jellyfish’s environment and consequently of jellyfish’s microbiome.

### Bacterial community composition of the various jellyfish body parts

Bacterial community composition differed between different *Aurelia* medusa body parts, especially the one within the gastral cavity (ANOSIM, global R = 0.53, p< 0.05) (Fig 2A). The communities of exumbrella and oral arms shared bacterial groups *Alphaproteobacteria* and *Gammaproteobacteria*, while the community in the gastral cavity was dominated by *Betaproteobacteria*, followed by *Gammaproteobacteria* and *Actinobacteria* (Fig 1). Within *Alphaproteobacteria*, bacterial communities of the exumbrella surface and oral arms were affiliated with *Phaeobacter*, *Ruegeria*, and within *Gammaproteobacteria* with *Stenotrophomonas*, *Alteromonas*, *Pseudoalteromonas*, and *Vibrio*. The community of oral arms was more diverse (S5 Table). In the gastral cavity were members of *Betaproteobacteria* affiliated with *Burkholderia*, *Cupriavidus*, and *Achromobacter*, of *Gammaproteobacteria* with *Pseudomonas*, and of *Actinobacteria* with *Kocuria* (Fig 2A, S1 Table).

**Fig 3:**
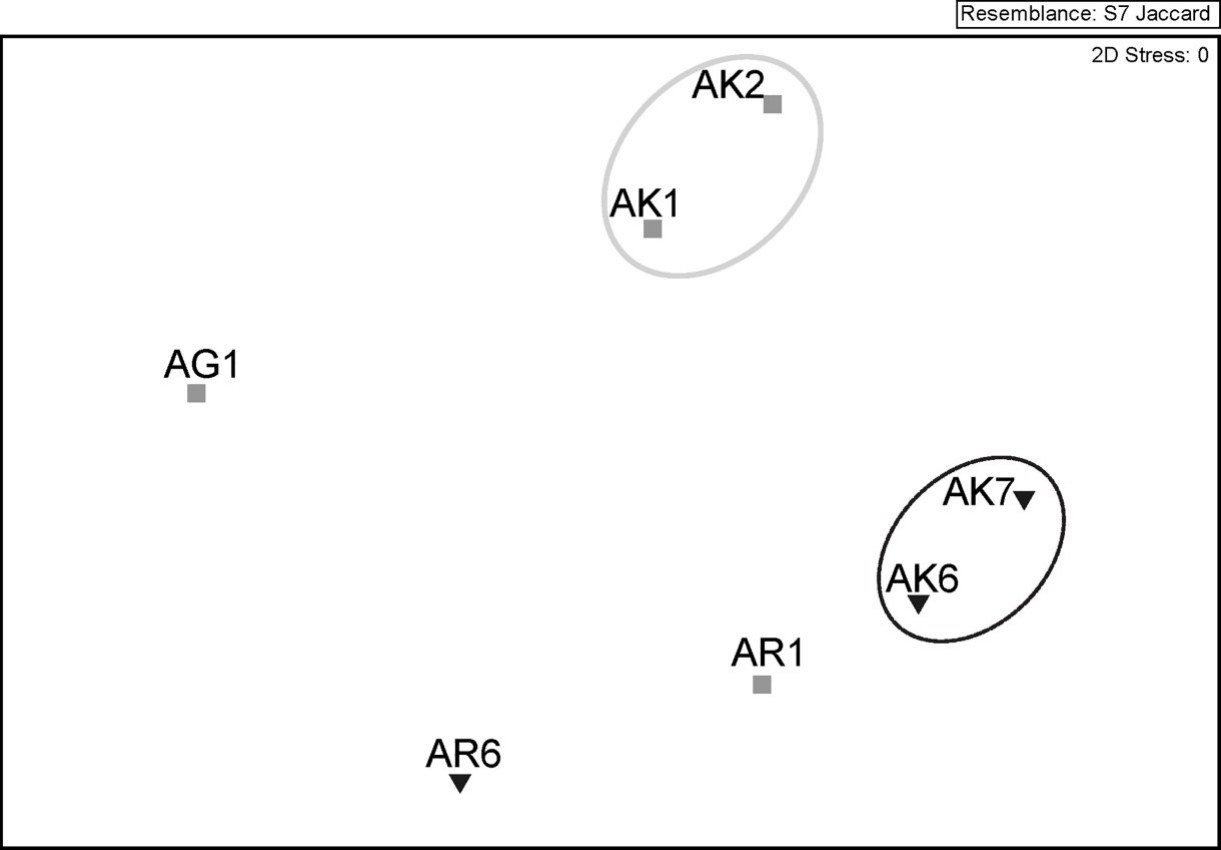
Non-metric multidimensional (nMDS) analysis based on bacterial community DGGE banding patterns of *Aurelia* jellyfish samples. AK- jellyfish exumbrella surface, AR-jellyfish oral arms, AG- mucus from gastral cavity. Samples were collected in May (grey squares) and June (inverted black triangles). Resemblance circles: grey line—40% similarity; black line—50% similarity.

The results of bacterial 16S rRNA gene clone libraries were also confirmed by DGGE-based non-metric multidimensional scaling (nMDS) analysis, since bacterial communities clustered together according to jellyfish body parts (Fig 3) (ANOSIM, global R = 0.63, p< 0.05). Phylogenetic information obtained from excised DGGE bands showed that bacterial taxa mostly belonged to *Alphaproteobacteria* (*Roseobacter*, *Phaeobacter*, *Ruegeria* all *Rhodobacteraceae*), but also *Gammaproteobacteria* (*Vibrio*, *Pseudoalteromonas*, *Stenotrophomonas*), and *Betaproteobacteria* (*Burkholderia*) (S1 Fig, S7 Table).

The exumbrella and oral arms surfaces are in constant contact with bacteria in the surrounding ambient seawater, attracted by secreted mucus, which is potentially a high-quality energy source and settling niche [84]. The bacteria of genus *Phaeobacter* and *Ruegeria*, are members of the *Roseobacter* clade, known as the successful surface colonizers, and utilizers of nutrients in the marine environment [84]. They produce acylated homoserine lactons (AHLs), the quorum-sensing signals involved in biofilm formation and function [84]. Bacteria of the *Pseudoalteromonas* genus produce extracellular enzymes and exopolysaccharides, which all together enable them to successfully compete for nutrients and colonization of surfaces [85]. Bacteria of *Alteromonas* and *Vibrio* genus are widespread in the marine environment and are common surface and particle colonizers [86]. According to Allers *et al*. [87], their versatile metabolism helps them exploit a complex substrate source, such as coral mucus, which in composition resembles to mucus produced by jellyfish *A*. *aurita* [62]. *Vibrio* species are major chitin utilizers, largely contributing to global carbon and nitrogen cycling. Although association with insoluble chitinous surface of detritus and live zooplankton is a preferable lifestyle for vibrios [86], they were found in association with other marine animals, including jellyfish, and are enriched in the seawater at the end of the jellyfish blooms [25]. *Vibrio coralliilyticus* was found in high abundances in coral tissue slurry [38], and proven to infect and cause tissue damage in corals at higher temperatures [88]. The *Stenotrophomonas* genus was usually represented in low abundances in communities associated with marine animals [32,34,38], but found to be producing antimicrobial compounds [89] and to be resistant to heavy metals and to degrade pollutants like polycyclic aromatic hydrocarbons (PAHs) and xenobiotics [90].

The dominance of *Betaproteobacteria* in medusa gastral cavity detected within our study is somehow surprising, since they are more characteristic for organic aggregates in limnetic ecosystems [91]. However, bacteria of the *Burkholderia* and *Achromobacter* genera were also isolated from the marine environment, including animals [92,93]. Among other characteristics, both were found to be able to degrade PAHs and to be resistant to multiple antibiotics [92–96]. Similarly, *Achromobacter* species were found to be n- alkane degrader and to remove also anthracene, phenanthrene, and pyrence from the environment [95]. The *Cupriavidus* species were not detected in the marine environment, to our knowledge, however, they were attributed with the ability to degrade aliphatic hydrocarbons [97]. Similarly, the marine *Pseudomonas* species were found to be able to degrade hydrocarbons like naphthalene, present within petroleum [96,98]. However, they were also found in association with sponges, producing antimicrobial compounds [31,89]. *Kocuria* isolated from marine sponges were found to produce the antibiotic kocurin [99,100] and to utilize polyethylene as a sole carbon source [101].

Based on our results we can only speculate what is the function of bacteria found in association with *Aurelia* sp. within our study. Hosts are supposed to recruit bacteria which are beneficial for their well-being. Strains of *Phaeobacter*, *Ruegeria*, *Pseudoalteromonas* and *Vibrio*, which we detected on exumbrella and oral arms surface, were previously recognized as important players in host defense against pathogens and fouling organisms from surrounding seawater [42,102–104], because of their ability to produce antimicrobial compounds when attached to live or inert surfaces [63,103,105–107]. However, more intriguing is the presence of *Burkholderia*, *Achromobacter and Kocuria* in the gastral cavity, since those bacteria have the ability to degrade PAH’s, xenobiotics, and plastic. Jellyfish mucus was found to have structural properties to effectively accumulate nanoparticles [59] and PAHs [108], which could be also transferred by ciliary currents and boundary layer flow to a marginal umbrella groove, and then to gastral cavity, since this is one type of prey capture recognized for *A*. *aurita* [109]. This could explain high abundances of hydrocarbon degrading bacteria found in the gut of *Aurelia* jellyfish within our study.

PAHs were found to be highly toxic for zooplankton organisms, however, adult medusa *A*. *aurita* and *M*. *leidyi* showed a high tolerance to exposure [110]. *A*. *aurita* under stress conditions, release blobs of mucus [60], detected also under exposure to crude oil (containing PAHs) [108]. In addition, when PAHs were entrapped within jellyfish mucus, hydrocarbon-degrading bacteria cell densities doubled, which resulted in an increased degradation of oil [108].

The Gulf of Trieste, an ecosystem where *Aurelia* was collected, is known to be polluted with PAHs (since Trieste and Koper are the main ports in the northern Adriatic) and other chemical compounds as well as by fecal bacteria, originating from coastal run off and municipal wastewater discharges [111]. This suggests that the bacterial community associated with jellyfish from this environment could be adapted to such conditions. Furthermore, supporting our hypothesis, polyps generating *Aurelia* medusa were found attached to port pillars [68]. This indicates that pollution adapted bacterial community could evolve and prosper at the polyp and medusa stages.

### Bacterial community structure shifts in *Aurelia* post-bloom period

Changes in jellyfish-associated bacterial communities, due to jellyfish population senescence, were evident in higher abundance of *Gammaproteobacteria* (Fig 1, S5 Table). SIMPER analysis showed that *Rhodobacteriaceae* and *Comamonadaceae* were relatively more abundant in the bacterial community associated with jellyfish at the peak of the bloom, while *Rhodobacteriaceae*, *Vibrionaceae*, and *Alteromonadaceae* in the bacterial community associated with senescent jellyfish (Table 1).

**Table 1:** Similarities percentage (SIMPER) analysis of 16S rRNA gene clone libraries from jellyfish samples collected at the time of population peak and at the end of the bloom in the Gulf of Trieste.

***Group May***						
Average similarity: 37,85						
**Species**	Av.Abund		Av.Sim	Sim/SD	Contrib%	Cum.%
*Rhodobacteraceae*	11.67		57.05	9.76	86.57	86.57
*Comamonadaceae*	0.67		1.9	0.58	2.89	89.46
*Alteromonadaceae*	1		1.9	0.58	2.89	92.35
***Group June***						
Average similarity: 73,40						
**Species**		Av.Abund	Av.Sim	Sim/SD	Contrib%	Cum.%
*Rhodobacteraceae*		10	32.55	2.15	44.34	44.34
*Vibrionaceae*		4.67	19.81	5.99	26.99	71.34
*Alteromonadaceae*		3.67	13.7	12.56	18.66	90
*Pseudoalteromonadaceae*		1.67	5.95	2.89	8.11	98.11
***Groups May & June***						
Average dissimilarity = 53,98						
**Species**	Group May Av.Abund	Group June Av.Abund	Av.Diss	Diss/SD	Contrib%	Cum.%
*Vibrionaceae*	1	4.67	9.01	2.83	22.63	22.63
*Rhodobacteraceae*	11.67	10	7.19	0.89	18.07	40.7
*Alteromonadaceae*	1	3.67	6.16	2.4	15.48	56.18
*Pseudoalteromonadaceae*	1	1.67	3.74	2.93	9.4	65.58
*Burkholderiaceae*	1.67	0.33	3.44	1	8.64	74.22
*Xanthomonadaceae*	1	0.33	2.17	1.16	5.45	79.67
*Pseudomonadaceae*	0.67	0.33	1.79	1	4.5	84.17
*Comamonadaceae*	0.67	0	1.7	1.32	4.27	88.44
*Hyphomicrobiaceae*	0.67	0	1.68	0.66	4.21	92.65

The major difference between both studied months was a rise in the temperature, which was higher in June, and the viability state of *Aurelia* jellyfish in the Gulf of Trieste. In June was the end of blooming period and jellyfish were in the decaying phase, which was evident as typical signs of moribund jellyfish [112]: degenerated tentacles, oral structures, and gonads, reduced swimming ability and necrosis of the epithelial bell tissue. This process is normally triggered by environmental stress like change in temperature or salinity, food availability, parasitism, and spawning, or even more likely, interacting stressors [112].

Jellyfish carcasses were found to be high quality labile organic substrate for bacteria [25]. Previous experiments following bacterial degradation of *Aurelia* jellyfish in the Gulf of Trieste, resulted in the increase of *Vibrio*’s relative abundance in the ambient microbial community [25]. In our study, *Vibrio* was associated with jellyfish at time of population peak and its relative abundance increased on senescent jellyfish at the end of the blooming period. This could suggest that vibrios are exploiting the nutrient-rich niche provided by *Aurelia*. Under the right conditions, like disturbed defense mechanism of jellyfish and higher temperature in the environment (documented in June in the Gulf of Trieste), up-regulating Vibrio’s virulence determinants such as motility, resistance to antimicrobial compounds, hemolysis, and cytotoxicity [86], they can outcompete other bacterial associates and become highly dominant.

Within our study, we consistently experienced unsuccessful amplification of bacterial 16S rRNA gene from jellyfish samples, unless additional PCR reaction was performed. Similar methodological problems with DNA amplification were reported before in the analyses of the tissue of healthy corals, and were attributed to the low abundance of bacterial associates [113], confirming previous observation of rare isolated bacterial cells within coral tissue by *in situ* hybridization [114]. The observed low number of *Aurelia* jellyfish-associated bacteria was also indicated by scanning electron microscopy of the medusa surface in our parallel study (data not shown). Our results from this study show that the surface was covered with mucus in the form of flocs, but no bacteria were noted at the epidermal surface. Using the same microscopic method, Johnston and Rohwer [115] similarly found that external cell layers of coral are invariably clean of adhering microbes, and they suggested the possibility of a dynamic community hovering in the boundary layers above the coral epidermis. This is also in agreement with observations by Weiland-Bräuer *et al*. [54], detecting the majority of bacteria located on the outer surface of coating mucus, covering *A*. *aurita* polips. The presence of rare bacterial cells could be due to the fact, that adult medusa has evolved mechanisms of defense against epibiotic organisms. One type of mechanism could be the production of antibacterial peptide aurelin, extracted from mesoglea of *A*. *aurita* [61]. It is also known that jellyfish surfaces, including *A*. *aurita*, are covered by a constantly renewing mucus layer, which was found to have implications in surface cleaning and defense against predators [58–60]. Similarly, as Garren and Azam [116] demonstrated for corals, surface mucus production could regulate an abundance of bacterial associates.

### Culturable bacteria

**Fig 4:**
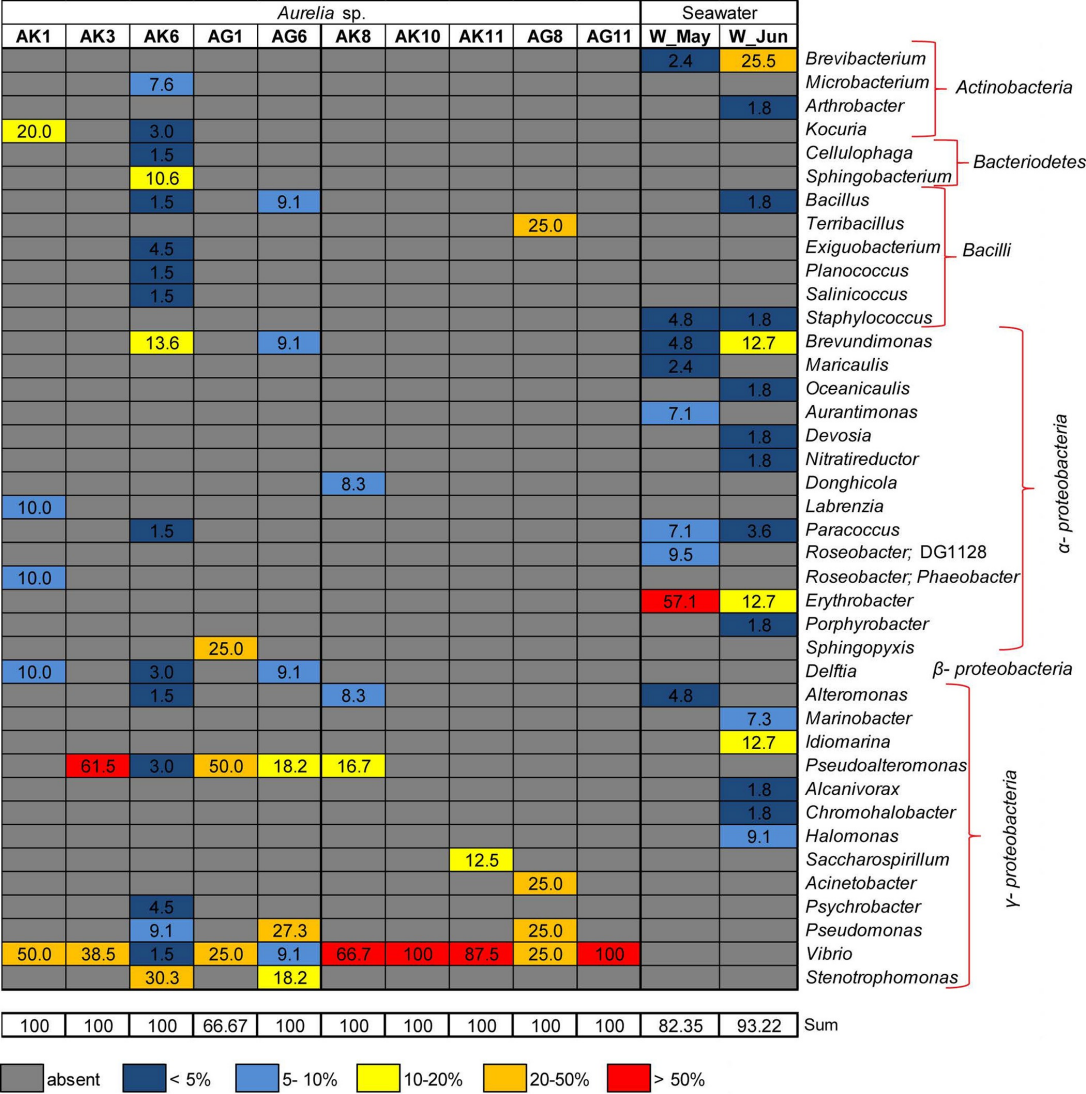
Bacterial isolates obtained from *Aurelia* jellyfish and ambient seawater. Heat map displaying relative abundance of bacterial genera across samples. Bacterial isolates obtained from jellyfish exumbrella surface (AK1, AK3, AK6), gastral cavity (AG1, AG6) and seawater (W_May) in May in the Gulf of Trieste.

Identification of 16S rRNA gene sequences of bacterial isolates revealed that bacteria predominantly belonged to *Gammaproteobacteria*, *Alphaproteobacteria*, *Betaproteobacteria*, *Actinobacteria*, *Bacteriodetes*, and *Firmicutes* (*Bacilli)*. Considering the main representatives within *Gammaproteobacteria*, *Vibrio* (*Vibrionaceae*), *Pseudoalteromonas* (*Pseudoalteromonadaceae*), and *Stenotrophomonas* (*Xanthomonadaceae*) dominated, but also *Pseudomonas* (*Pseudomonadaceae*), *Alteromonas* (*Alteromonadaceae*), and *Psyhrobacter* (*Moraxellaceae*) were detected. Representatives of *Alphaproteobacteria* were mostly *Labrenzia* and *Phaeobacter* (*Rhodobacteraceace*), and of *Actinobacteia* mostly *Kocuria* (*Micrococcaceae*) and *Microbacterium* (*Microbacteriaceae*) (Fig 4, S2 Table).

The differences were observed between culturable part of seawater and jellyfish-associated communities (ANOSIM, global R = 0.92, p< 0.05), with seawater communities being more diverse (Fig 4, S2 Table and S6 Table). According to SIMPER analysis, *Erythrobacter*, *Brevibacterium*, and *Brevundimonas*, characteristic taxa in culturable fraction of water assemblages, contributed the most to the difference between bacteria isolated from jellyfish and water (S4 Table).

Bacteria isolated from senescent jellyfish, exumbrella and gastral cavity, mostly belonged to *Gammaproteobacteria* (Fig 4B) and within *Vibrio* became highly dominant representative (Fig 4B, S2 Table). The community composition change was evident in lower diversity (S6 Table) and dominance of *Vibrio* was also confirmed by SIMPER analysis (S8 Table).

Considering the main representatives within bacterial groups, 16S rRNA clone libraries and the culture-dependent method more or less pointed to the presence of the same bacterial taxa. With the exception of *Ruegeria*, *Burkholderia*, *Cupriavidus and Achromobacter*, other bacteria were also recovered by culturing. In addition, the culturing approach revealed the presence of bacteria affiliated with *Microbacterium* (*Microbacteriaceae*), *Sphingobacterium* (*Sphingobacteriaceae*), *Brevundimonas* (*Caulobacteraceae*), and *Delftia* (*Comamonadaceae*), which were not detected within clone libraries.

## Conclusion

Both culture-dependent and independent methods have been extensively used to study and to understand the role of microbial communities associated with marine animals, especially crustacean zooplankton, benthic sponges, and corals. Data available for *Aurelia*’s associated microbial community is still limited and needs to be further examined. With the exception of *Mycoplasma* bacteria, a possible endosymbiont detected within *A*. *aurita* tissue [46,54], the nature of the relationship between *Aurelia* jellyfish and bacterial associates is not straight forward. In addition, it is hard to say whether these bacteria are true residents of jellyfish, forming a species-specific association with the host, or are they just opportunistic microbes residing in a niche of an organically-rich environment. So far, we can only speculate on the role of bacterial associates, although they may play important functional roles during the life cycle of *A*. *aurita* [54].

Our results showed that bacterial community associated with *Aurelia* jellyfish in the Gulf of Trieste is distinct from ambient seawater assemblage and differ between medusa body parts. Our results suggest that associated bacteria could be host- promoted and that anthropogenic pressure, present in the Gulf of Trieste, could play a role in structuring the associated communities. However, clone library sequencing depth available in our study is a limitation for drawing conclusions about the community stability and deeper sequencing would allow a more detailed assessment of indicated differences.

Further investigation of jellyfish–bacteria relationship is required to understand the relevance of the associated bacteria for the host during its life span and during/after the bloom period, especially in areas facing seasonal blooms, which influence food webs, biogeochemical cycles and their impact on ecosystem.

Finally, we would also like to emphasize the importance of culturing organisms. The culture-based studies are again gaining attention and recognition for helping us to better integrate the physiological, ecological and genomic-based information [117]. Although the method is biased towards certain bacterial groups, our results based on culture methods are in line with results from a culture-independent approach. In addition, our collection of bacterial isolates might be important to obtain complete genome sequences, to study physiology and biochemistry of specific bacteria, to help understand the biology and ecology, as well as to exploit their biotechnological potential.

## Supporting information

S1 Table Composition of 16S rRNA gene clone libraries (% of clones) from samples of jellyfish exumbrella (*AK*), oral arms (*AR*), and mucus from gastral cavity (*AG)* and seawater samples (*W*) at 5m depth collected on May and June 2011 in the Gulf of Trieste. Classification of bacterial clones was done down to the family level. The contribution of distinct bacterial taxa is expressed as a percentage of the total number of sequences in each sample. *N* is the total number of bacterial clones in the library. Numbers (*N*) in light grey are the total number of sequences recovered from clone library, including sequences affiliated with Chloroplast (%; presented at the bottom of table).
(PDF)

S2 Table Bacterial isolates obtained from samples of jellyfish exumbrella (*AK*) and mucus from gastral cavity (*AG*) and seawater samples (*W*) at 5 m depth collected on May and June 2011 in the Gulf of Trieste. Classification of bacterial isolates was done down to the genus level. The contribution of distinct bacterial taxa is expressed as a percentage of the total number of sequences in each sample. *N* is the total number of isolated bacteria.
(PDF)

S3 Table Similarities percentage (SIMPER) analysis of 16S rRNA gene clone libraries from samples of jellyfish exumbrella (*AK*), oral arms (*AR*), and mucus from gastral cavity (*AG*) and seawater samples (*W*) collected on May and June 2011 in the Gulf of Trieste. (PDF)

S4 Table Similarities percentage (SIMPER) analysis of culturable fraction of the bacterial community associated with jellyfish exumbrella (*AK*), mucus from gastral cavity (*AG*) and seawater (*W*) collected in May and June 2011 in the Gulf of Trieste. (PDF)

S5 Table The diversity indices S, H’, d, J’, Chao- 1 and library coverage’s (*C*) describing composition of total bacterial community associated with jellyfish exumbrella (*AK)*, oral arms (*AR*) and mucus from gastral cavity (*AG*) and seawater (*W*) collected in May and June 2011 in the Gulf of Trieste. *S* represents the number of distinct bacterial taxa detected in each bacterial 16S rRNA gene clone library. *C* is a coverage value (*C* = (1–n1/N), where *n1* is number of phylotypes appearing only once in the library and *N* is the library size.
(PDF)

S6 Table The diversity indices S, H’, d, J’, describing composition of culturable fraction of bacterial community associated with jellyfish exumbrella (*AK*) and mucus from gastral cavity (*AG*) and seawater (*W)* collected in May and June 2011 in the Gulf of Trieste. *S* represents the number of distinct bacterial taxa detected in each sample.
(PDF)

S7 Table Bacterial 16S rRNA sequences obtained from DGGE bands from jellyfish samples with their accession numbers. In the table is the name and an accession number of their closest relative in GeneBank (NCBI) with % of similarity, family, taxon, and isolation source.
(PDF)

S8 Table Similarities percentage (SIMPER) analysis of culturable fraction of bacterial community associated with jellyfish at the time of population peak and at the end of the bloom in the Gulf of Trieste. Group May includes samples of jellyfish exumbrella surface (*AK1*, *AK3*, *AK6*) and gastral cavity (*AG1*, *AG6*) collected in May. Group June includes samples of jellyfish exumbrella surface (*AK8*, *AK10*, *AK11*) and gastral cavity (*AG8*, *AG11*) collected in June.
(PDF)

S1 Fig DGGE profile of bacterial 16S rRNA gene fragments of samples from *Aurelia* jellyfish exumbrella surface, oral arms, and mucus from gastral cavity. *AK1*, *AK2*: exumbrella surface of jellyfish collected in May; *AK6*, *AK7*: the exumbrella surface of jellyfish collected in June; *AR1*: sample of oral arms of jellyfish collected in May; *AR6*: the oral arms of jellyfish collected in June; *AG1*: the gastral cavity mucus sample; *S*: standard. The numbers on the figure represent bands that were cut from the gel and successfully sequenced; color dots place sequence in one of the bacterial groups.
(PDF)

S1 Protocol Detailed description of DNA extraction methods, with modifications. (PDF)

S2 Protocol PCR reaction mix composition and PCR temperature cycling conditions. (PDF)
