# Supplementary material for: Bacteria associated with moon jellyfish during bloom and post-bloom periods in the Gulf of Trieste (northern Adriatic)
Source: PLoS One. 2019 Jan 15;14(1):e0198056. doi: 10.1371/journal.pone.0198056 (PMC6333360; doi:10.1371/journal.pone.0198056)
Supplement: S2 Table — Classification of bacterial isolates was done down to the genus level. The contribution of distinct bacterial taxa is expressed as a percentage of the total number of sequences in each sample. N is the total number of isolated bacteria. (PDF) [file pone.0198056.s002.pdf]

S2 Table. Bacterial isolates obtained from samples of jellyfish exumbrella (AK) and mucus from gastral cavity (AG) and seawater samples (W) at 5 m depth collected on May and June 2011 in the Gulf of Trieste. Classification of bacterial isolates was done down to the genus level. The contribution of distinct bacterial taxa is expressed as a percentage of the total number of sequences in each sample. N is the total number of isolated bacteria.

				Aurelia Jellyfish										Seawater	
				May					June						
			Sample	**AK1**	**AK3**	**AK6**	**AG1**	**AG6**	**AK8**	**AK10**	**AK11**	**AG8**	**AG11**	**W_May**	**W_Jun**
Class	Family	Genus	N	10	13	66	6	11	12	7	8	4	4	51	59
Actinobacteria	*Brevibacteriaceae*	*Brevibacterium*		_	_	_	_	_	_	_	_	_	_	1.96	23.73
	Microbacteriaceae	*Microbacterium*		_	_	7.58	_	_	_	_	_	_	_	_	_
	Micrococcaceae	*Arthrobacter*		_	_	_	_	_	_	_	_	_	_	_	1.69
		Kocuria		20.00	_	3.03	_	_	_	_	_	_	_	_	_
Flavobacteria	*Flavobacteriaceae*	*Cellulophaga*		_	_	1.52	_	_	_	_	_	_	_	_	_
Sphingobacteria	*Sphingobacteriaceae*	*Sphingobacterium*		_	_	10.61	_	_	_	_	_	_	_	_	_
Bacilli	*Bacillaceae*	*Bacillus*		_	_	1.52	_	9.09	_	_	_	_	_	_	1.69
		Terribacillus		_	_	_	_	_	_	_	_	25.00	_	_	_
	Family_XII_Incertae_Sedis	*Exiguobacterium*		_	_	4.55	_	_	_	_	_	_	_	_	_
	Planococcaceae	*Planoccocus*		_	_	1.52	_	_	_	_	_	_	_	_	_
	Staphylococcaceae	*Salinicoccus*		_	_	1.52	_	_	_	_	_	_	_	_	_
		Staphylococcus		_	_	_	_	_	_	_	_	_	_	3.92	1.69
α-proteobacteria	*Caulobacteraceae*	*Brevundimonas*		_	_	13.64	_	9.09	_	_	_	_	_	3.92	11.86
	Hyphomonadaceae	*Maricaulis*		_	_	_	_	_	_	_	_	_	_	1.96	_
		Oceanicaulis		_	_	_	_	_	_	_	_	_	_	_	1.69
	Aurantimonadaceae	*Aurantimonas*		_	_	_	_	_	_	_	_	_	_	5.88	_
	Hyphomicrobiaceae	*Devosia*		_	_	_	_	_	_	_	_	_	_	_	1.69
	Phyllobacteriaceae	*Nitratireductor*		_	_	_	_	_	_	_	_	_	_	_	1.69
		Unclass. *Phyllobacteriaceae*		_	_	_	_	_	_	_	_	_	_	9.80	3.39
	Rhodobacteraceae	*Donghicola*		_	_	_	_	_	8.33	_	_	_	_	_	_
		Labrenzia		10.00	_	_	_	_	_	_	_	_	_	_	_
		Paracoccus		_	_	1.52	_	_	_	_	_	_	_	5.88	3.39
		Marivita		_	_	_	_	_	_	_	_	_	_	7.84	_
		Phaeobacter		10.00	_	_	_	_	_	_	_	_	_	_	_
		Unclass. *Rhodobacteraceae*		_	_	_	16.67	_	_	_	_	_	_	5.88	3.39
	Erythrobacteraceae	*Erythrobacter*		_	_	_	_	_	_	_	_	_	_	47.06	11.86
		Porphyrobacter		_	_	_	_	_	_	_	_	_	_	_	1.69
		Unclass. *Erythrobacteraceae*		_	_	_	_	_	_	_	_	_	_	_	_
	Sphingomonadaceae	*Sphingopyxis*		_	_	_	16.67	_	_	_	_	_	_	_	_
β-proteobacteria	*Comamonadaceae*	*Delftia*		10.00	_	3.03	_	9.09	_	_	_	_	_	_	_
		Unclass. *Comamonadaceae*		_	_	_	16.67	_	_	_	_	_	_	_	_
γ-proteobacteria	*Alteromonadaceae*	*Alteromonas*		_	_	1.52	_	_	8.33	_	_	_	_	3.92	_

	Marinobacter	_	_	_	_	_	_	_	_	_	_	_	6.78
Idiomarinaceae	*Idiomarina*	_	_	_	_	_	_	_	_	_	_	_	11.86
Pseudoalteromonadaceae	*Pseudoalteromonas*	_	61.54	3.03	33.33	18.18	16.67	_	_	_	_	_	_
Alcanivoracaceae	*Alcanivorax*	_	_	_	_	_	_	_	_	_	_	_	1.69
Halomonadaceae	*Chromohalobacter*	_	_	_	_	_	_	_	_	_	_	_	1.69
	Halomonas	_	_	_	_	_	_	_	_	_	_	_	8.47
Saccharospirillaceae	*Saccharospirillum*	_	_	_	_	_	_	_	12.50	_	_	_	_
Moraxellaceae	*Acinetobacter*	_	_	_	_	_	_	_	_	25.00	_	_	_
	Psychrobacter	_	_	4.55	_	_	_	_	_	_	_	_	_
Pseudomonadaceae	*Pseudomonas*	_	_	9.09	_	27.27	_	_	_	25.00	_	_	_
Vibrionaceae	*Vibrio*	50.00	38.46	1.52	16.67	9.09	66.67	100.00	87.50	25.00	100.00	_	_
Xanthomonadaceae	*Stenotrophomonas*	_	_	30.30	_	18.18	_	_	_	_	_	_	_
Unclass. *Gammaproteobacteria*		_	_	_	_	_	_	_	_	_	_	1.96	_
	Total	100	100	100	100	100	100	100	100	100	100	100	100
