# Supplementary material for: Bacteria associated with moon jellyfish during bloom and post-bloom periods in the Gulf of Trieste (northern Adriatic)
Source: PLoS One. 2019 Jan 15;14(1):e0198056. doi: 10.1371/journal.pone.0198056 (PMC6333360; doi:10.1371/journal.pone.0198056)
Supplement: S3 Table — (PDF) [file pone.0198056.s003.pdf]

S3 Table. Similarities percentage (SIMPER) analysis of 16S rRNA gene clone libraries from samples of jellyfish exumbrella (AK), oral arms (AR), and mucus from gastral cavity (AG) and seawater samples (W) collected on May and June 2011 in the Gulf of Trieste.

Group A*

Average similarity: 47,60

Species	Av.Abund	Av.Sim	Sim/SD	Contrib%	Cum.%
Rhodobacteraceae	9.29	31.83	1.33	66.86	66.86
Vibrionaceae	2.43	4.99	0.71	10.48	77.35
Alteromonadaceae	2	4.42	0.82	9.29	86.64
Pseudoalteromonadaceae	1.14	1.85	0.59	3.89	90.52

* In group A are included all jellyfish samples

Group W*

Average similarity: 44,22

Species	Av.Abund	Av.Sim	Sim/SD	Contrib%	Cum.%
Synechococcus	6.33	11.44	7.23	25.88	25.88
Flavobacteriaceae	10	8.15	1.3	18.43	44.31
Litoricolaceae	3.33	4.3	7.58	9.73	54.04
SAR11	5.33	4.07	1.3	9.21	63.25
Microbacteriaceae	2.33	2.84	2.48	6.42	69.67
Cryomorphaceae	2	2.84	2.48	6.42	76.09
Rhodobacteraceae	4.33	2.84	2.48	6.42	82.51
Alteromonadaceae	3	2.15	7.58	4.86	87.37
Puniceicoccaceae	1	2.15	7.58	4.86	92.24

**Seawater group (W) includes water samples collected at 5m depth in May and June 2011 and in May 2010*

Groups A & W

Average dissimilarity = 84,79

Species	Group A Av.Abund	Group W Av.Abund	Av.Diss	Diss/SD	Contrib%	Cum.%
Flavobacteriaceae	0	10	14.29	1.22	16.85	16.85
Rhodobacteraceae	9.29	4.33	9.43	1.61	11.12	27.97
Synechococcus	0.29	6.33	8.71	4.35	10.27	38.24
SAR11	0	5.33	6.97	1.41	8.21	46.46
Litoricolaceae	0	3.33	4.91	1.63	5.79	52.24
SAR116	0	3.67	4.61	0.98	5.44	57.68
Burkholderiaceae	3	0	3.78	0.61	4.46	62.14
Alteromonadaceae	2	3	3.63	1.43	4.28	66.42
Vibrionaceae	2.43	0	3.59	1.12	4.23	70.66
Microbacteriaceae	0	2.33	3.53	1.5	4.16	74.82
Cryomorphaceae	0	2	2.97	1.9	3.5	78.32
Pseudomonadaceae	1.86	0	2.31	0.56	2.72	81.05
Pseudoalteromonadaceae	1.14	0	1.65	0.99	1.95	82.99
Chitinophagaceae	0	1	1.52	1.06	1.79	84.78
Puniceicoccaceae	0	1	1.46	5.99	1.72	86.5
SAR86	0	1	1.3	1.27	1.53	88.03
OCS116	0	1	1.2	0.69	1.42	89.45
