# Supplementary material for: Bacteria associated with moon jellyfish during bloom and post-bloom periods in the Gulf of Trieste (northern Adriatic)
Source: PLoS One. 2019 Jan 15;14(1):e0198056. doi: 10.1371/journal.pone.0198056 (PMC6333360; doi:10.1371/journal.pone.0198056)
Supplement: S4 Table — (PDF) [file pone.0198056.s004.pdf]

S4 Table. Similarities percentage (SIMPER) analysis of culturable fraction of the bacterial community associated with jellyfish exumbrella (AK), mucus from gastral cavity (AG) and seawater (W) collected in May and June 2011 in the Gulf of Trieste.

Groups A* & W**
Average dissimilarity = 98,21

Species	Group A Av.Abund	Group W Av.Abund	Av.Diss	Diss/SD	Contrib%	Cum.%
Erythrobacter	0	15.5	28.06	1.48	28.57	28.57
Brevibacterium	0	7.5	11.54	1.13	11.75	40.32
Brevundimonas	1	4.5	7.05	1.86	7.17	47.49
Vibrio	4	0	6.98	1.4	7.11	54.6
Idiomarina	0	3.5	5.29	0.95	5.39	59.99
Paracoccus	0.1	2.5	4.27	2.45	4.35	64.34
Roseobacter; DG1128	0	2	3.79	0.94	3.86	68.2
Halomonas	0	2.5	3.78	0.95	3.85	72.05
Marinobacter	0	2	3.03	0.95	3.08	75.13
Aurantimonas	0	1.5	2.85	0.94	2.9	78.03
Staphylococcus	0	1.5	2.65	2.06	2.7	80.73
Pseudoalteromonas	1.6	0	2.55	0.64	2.6	83.33
Stenotrophomonas	2.2	0	2.09	0.39	2.13	85.46
Alteromonas	0.2	1	1.87	0.99	1.91	87.37
Pseudomonas	1	0	1.23	0.58	1.25	88.62
Maricaulis	0	0.5	0.95	0.94	0.97	89.59
Bacillus	0.2	0.5	0.78	0.95	0.79	90.38

* In group A are included all jellyfish samples

**Seawater group (W) includes water samples collected at 5m depth in May and June.

Groups AK & AG
Average dissimilarity = 73,90

Species	Group AK Av.Abund	Group AG Av.Abund	Av.Diss	Diss/SD	Contrib%	Cum.%
Vibrio	5.5	1.75	26.1	1.5	35.32	35.32
Pseudoalteromonas	2	1	11.06	0.76	14.97	50.29
Stenotrophomonas	3.33	0.5	6.55	0.64	8.87	59.15
Pseudomonas	1	1	5.72	0.99	7.73	66.89
Brevundimonas	1.5	0.25	3.04	0.65	4.12	71.01
Kocuria	0.67	0	2.65	0.53	3.58	74.59
Delftia	0.5	0.25	2.11	0.78	2.85	77.44
Sphingobacterium	1.17	0	1.63	0.44	2.2	79.65
Terribacillus	0	0.25	1.59	0.52	2.15	81.8
Sphingopyxis	0	0.25	1.59	0.52	2.15	83.95
Acinetobacter	0	0.25	1.59	0.52	2.15	86.1
Saccharospirillum	0.17	0	1.26	0.43	1.71	87.8
Alteromonas	0.33	0	1.2	0.55	1.62	89.42
Bacillus	0.17	0.25	1.18	0.6	1.6	91.02
