# Supplementary material for: Bacteria associated with moon jellyfish during bloom and post-bloom periods in the Gulf of Trieste (northern Adriatic)
Source: PLoS One. 2019 Jan 15;14(1):e0198056. doi: 10.1371/journal.pone.0198056 (PMC6333360; doi:10.1371/journal.pone.0198056)
Supplement: S5 Table — S represents the number of distinct bacterial taxa detected in each bacterial 16S rRNA gene clone library. C is a coverage value (C = (1–n1/N), where n1 is number of phylotypes appearing only once in the library and N is the library size. (PDF) [file pone.0198056.s005.pdf]

S5 Table. The diversity indices S, H', d, J', Chao- 1 and library coverage's (C) describing composition of total bacterial community associated with jellyfish exumbrella (AK), oral arms (AR) and mucus from gastral cavity (AG) and seawater (W) collected in May and June 2011 in the Gulf of Trieste. S represents the number of distinct bacterial taxa detected in each bacterial 16S rRNA gene clone library. C is a coverage value ($C = (1–n_1/N)$, where n_1 is number of phylotypes appearing only once in the library and N is the library size.

	Sample	Species richness (S)	Shannon (H)	Margalef (d)	Equitability (J')	Chao-1	Coverage value (C; %)	N	n_1
May	AK1	8	1.49	2.30	0.72	11.33	0.76	21	5
	AK2	8	1.59	2.30	0.76	9.5	0.81	21	4
	AR1	11	2.03	2.86	0.85	12.5	0.88	33	4
	AG1	8	1.74	1.86	0.83	8.5	0.95	43	2
	W_May	16	1.95	3.43	0.70	17.67	0.94	79	5
June	AK6	7	1.61	1.80	0.83	7	0.96	28	1
	AK7	9	1.86	2.25	0.85	10	0.91	35	3
	AR6	13	2.36	3.60	0.92	23.5	0.75	28	7
	W_June	23	2.76	5.08	0.88	27.5	0.88	76	9
