# Supplementary material for: Bacteria associated with moon jellyfish during bloom and post-bloom periods in the Gulf of Trieste (northern Adriatic)
Source: PLoS One. 2019 Jan 15;14(1):e0198056. doi: 10.1371/journal.pone.0198056 (PMC6333360; doi:10.1371/journal.pone.0198056)
Supplement: S6 Table — S represents the number of distinct bacterial taxa detected in each sample. (PDF) [file pone.0198056.s006.pdf]

S6 Table. The diversity indices S, H’, d, J’ describing composition of culturable fraction of bacterial community associated with jellyfish exumbrella (AK) and mucus from gastral cavity (AG) and seawater (W) collected in May and June 2011 in the Gulf of Trieste. S represents the number of distinct bacterial taxa detected in each sample.

	Sample	Species richness (S)	Shannon (H)	Margalef (d)	Equitability (J')
May	AK1	5	1.36	1.74	0.84
	AK3	2	0.67	0.39	0.96
	AK6	17	2.33	3.82	0.82
	AG1	5	1.56	2.23	0.97
	AG6	7	1.85	2.50	0.95
	W_May	12	1.89	2.80	0.76
June	AK8	4	0.98	1.21	0.71
	AK10	1	0	0	0
	AK11	2	0.38	0.48	0.54
	AG8	4	1.39	2.16	1.00
	AG11	1	0	0	0
	W_June	18	2	4	0.8504
