# Supplementary material for: Bacteria associated with moon jellyfish during bloom and post-bloom periods in the Gulf of Trieste (northern Adriatic)
Source: PLoS One. 2019 Jan 15;14(1):e0198056. doi: 10.1371/journal.pone.0198056 (PMC6333360; doi:10.1371/journal.pone.0198056)
Supplement: S7 Table — In the table is the name and an accession number of their closest relative in GeneBank (NCBI) with % of similarity, family, taxon, and isolation source. (PDF) [file pone.0198056.s007.pdf]

S7 Table. Bacterial 16S rRNA equences obtained from DGGE bands from jellyfish samples with their accession numbers. In the table there is also the name and an accession number of their closest relative in GeneBank (NCBI) with % of similarity, family, taxon and isolation source.

DGGE Band	Acc. No.	Class	Family	Genus	Closest relative in GeneBank; Acc. No.	Similarity [%]	Isolation source
BAND_A02_AK2_1	MF952749	*Alphaproteobacteria*	*Rhodobacteraceae*	*Ruegeria*	Ruegeria sp. S11AQ 16S ribosomal RNA gene, partial sequence; KF188478	100	Host: sponge *Asbestopluma hypogea*
BAND_A03_AK6_1	MF952750	*Deinococci*	*Trueperaceae*	*Truepera*	Uncultured bacterium clone THERM1 16S ribosomal RNA gene, partial sequence; AY494700	98	Salmo salar gill
BAND_A04_AR1_1	MF952751	*Gammaproteobacteria*	*Vibrionaceae*	*Vibrio*	Uncultured bacterium clone SanDiego_a2803 16S ribosomal RNA gene, partial sequence; KF799588	97	Ocean water
BAND_B01_AK1_1	MF952752	*Alphaproteobacteria*	*Rhodobacteraceae*	*Roseobacter*	Roseobacter sp. Z7 16S ribosomal RNA gene, partial sequence; KT461667	98	Host: brown alga *Ectocarpus* sp.
BAND_B04_AR6_1	MF952753	*Betaproteobacteria*	*Burkholderiaceae*	*Burkholderia*	Uncultured Burkholderia sp. gene for 16S ribosomal RNA, partial sequence clone: LR564B-55; LC001458	98	Lake
BAND_C02_AK2_2	MF952754	*Actinobacteria*	*Coriobacteriaceae*	*Atopobium*	Uncultured bacterium clone ncd1381f11c1 16S ribosomal RNA gene, partial sequence; JF120828	97	Skin, popliteal fossa
BAND_C03_AK7_1	MF952755	*Gammaproteobacteria*	*Vibrionaceae*	*Vibrio*	Vibrio cyclitrophicus strain Mj149 16S ribosomal RNA gene, partial sequence; GQ454943	99	Hemolymph of spider crab
BAND_D03_AK7_2	MF952756	*Betaproteobacteria*	*Comamonadaceae*	*Pelomonas*	Uncultured beta proteobacterium gene for 16S rRNA, partial sequence, isolate: DGGE band: OBmeso04-51; AB367341	99	Coastal seawater
BAND_D04_AR6_2	MF952757	*Gammaproteobacteria*	*Pseudoalteromonadaceae*	*Pseudoalteromonas*	Uncultured Pseudoalteromonas sp. clone JL-BS-K65 16S ribosomal RNA gene, partial sequence; AY664354	99	Sea water
BAND_E01_AK1_2	MF952758	*Alphaproteobacteria*	*Rhodobacteraceae*		Uncultured bacterium clone H9pH79a 16S ribosomal RNA gene, partial sequence; JQ179029	98	Host: crustose coralline algae
BAND_E03_AK7_3	MF952759	*Actinobacteria*	*Microbacteriaceae*	*Leucobacter*	Leucobacter sp. C7.oil.3 16S ribosomal RNA gene, partial sequence; KJ756157	97	Gulf of Mexico sediment
BAND_E04_AR6_3	MF952760	*Gammaproteobacteria*	*Xanthomonadaceae*	*Stenotrophomonas*	Uncultured bacterium clone DolGs_A056 16S ribosomal RNA gene, partial sequence; JQ194688	97	Deep-sea sediments
BAND_F01_AK1_3	MF952761	*Alphaproteobacteria*	*Rhodobacteraceae*	*Phaeobacter*	Phaeobacter sp. PP_B2A.4 16S ribosomal RNA gene, partial sequence; KC250896	99	Host: diatom *Pseudo-nitzschia pungens*
BAND_G04_AG1_1	MF952762	*Gammaproteobacteria*	*Xanthomonadaceae*	*Stenotrophomonas*	Stenotrophomonas maltophilia strain KW 98 16S ribosomal RNA gene, partial sequence;JX262398	97	Arctic ocean sediment and water
BAND_H01_AK1_4	MF952763	*Gammaproteobacteria*	*Xanthomonadaceae*	*Stenotrophomonas*	Stenotrophomonas maltophilia strain ISMMS2, complete genome; CP011305	98	Complete genome
