# Supplementary material for: Bacteria associated with moon jellyfish during bloom and post-bloom periods in the Gulf of Trieste (northern Adriatic)
Source: PLoS One. 2019 Jan 15;14(1):e0198056. doi: 10.1371/journal.pone.0198056 (PMC6333360; doi:10.1371/journal.pone.0198056)
Supplement: S8 Table — Group May includes samples of jellyfish exumbrella surface (AK1, AK3, AK6) and gastral cavity (AG1, AG6) collected in May. Group June includes samples of jellyfish exumbrella surface (AK8, AK10, AK11) and gastral cavity (AG8, AG11) collected in June. (PDF) [file pone.0198056.s008.pdf]

**S8 Table. Similarities percentage (SIMPER) analysis of culturable fraction of bacterial community associated with jellyfish at the time of population peak and at the end of the bloom in the Gulf of Trieste.** Group May includes samples of jellyfish exumbrella surface (AK1, AK3, AK6) and gastral cavity (AG1, AG6) collected in May. Group June includes samples of jellyfish exumbrella surface (AK8, AK10, AK11) and gastral cavity (AG8, AG11) collected in June.

Group May
Average similarity: 23,24

| Species | Av.Abund | Av.Sim | Sim/SD | Contrib% | Cum.% |
|---|---|---|---|---|---|
| *Vibrio* | 2.6 | 11.13 | 0.91 | 47.91 | 47.91 |
| *Pseudoalteromonas* | 2.8 | 8.28 | 0.81 | 35.65 | 83.56 |
| *Delftia* | 0.8 | 1.48 | 0.49 | 6.35 | 89.91 |
| *Pseudomonas* | 1.8 | 0.78 | 0.32 | 3.35 | 93.26 |

Group June
Average similarity: 49,88

| Species | Av.Abund | Av.Sim | Sim/SD | Contrib% | Cum.% |
|---|---|---|---|---|---|
| *Vibrio* | 5.4 | 49.88 | 1.7 | 100 | 100 |

Groups May & June
Average dissimilarity = 72,88

| Species | Group May Av.Abund | Group June Av.Abund | Av.Diss | Diss/SD | Contrib% | Cum.% |
|---|---|---|---|---|---|---|
| *Vibrio* | 2.6 | 5.4 | 18.56 | 1.16 | 25.46 | 25.46 |
| *Pseudoalteromonas* | 2.8 | 0.4 | 13.98 | 0.91 | 19.19 | 44.65 |
| *Stenotrophomonas* | 4.4 | 0 | 7.77 | 0.7 | 10.66 | 55.31 |
| *Pseudomonas* | 1.8 | 0.2 | 5.76 | 0.89 | 7.91 | 63.22 |
| *Brevundimonas* | 2 | 0 | 3.61 | 0.72 | 4.95 | 68.17 |
| *Kocuria* | 0.8 | 0 | 2.97 | 0.61 | 4.08 | 72.25 |
| *Delftia* | 0.8 | 0 | 2.9 | 1.05 | 3.98 | 76.23 |
| *Sphingopyxis* | 0.2 | 0 | 1.95 | 0.47 | 2.67 | 78.9 |
| *Sphingobacterium* | 1.4 | 0 | 1.92 | 0.49 | 2.64 | 81.53 |
| *Bacillus* | 0.4 | 0 | 1.41 | 0.62 | 1.94 | 83.47 |
| *Microbacterium* | 1 | 0 | 1.37 | 0.49 | 1.88 | 85.36 |
| *Terribacillus* | 0 | 0.2 | 1.34 | 0.42 | 1.85 | 87.2 |
| *Acinetobacter* | 0 | 0.2 | 1.34 | 0.42 | 1.85 | 89.05 |
| *Labrenzia* | 0.2 | 0 | 1.21 | 0.48 | 1.66 | 90.71 |
