# Supplementary material for: Bacteria associated with moon jellyfish during bloom and post-bloom periods in the Gulf of Trieste (northern Adriatic)
Source: PLoS One. 2019 Jan 15;14(1):e0198056. doi: 10.1371/journal.pone.0198056 (PMC6333360; doi:10.1371/journal.pone.0198056)

S1 Fig. DGGE profile of bacterial 16S rRNA gene fragments of samples from *Aurelia* jellyfish exumbrella surface, oral arms and mucus from gastral cavity. AK1, AK2: the exumbrella surface sample of jellyfish collected in May; AK6, AK7: the exumbrella surface sample of jellyfish collected in June; AR1: sample of oral arms of jellyfish collected in May; AR6: sample of oral arms of jellyfish collected in June; AG1: the gastral cavity mucus sample; S: standard. The numbers on the figure represent bands that were cut from the gel and successfully sequenced; color dots place sequence in one of bacterial groups.

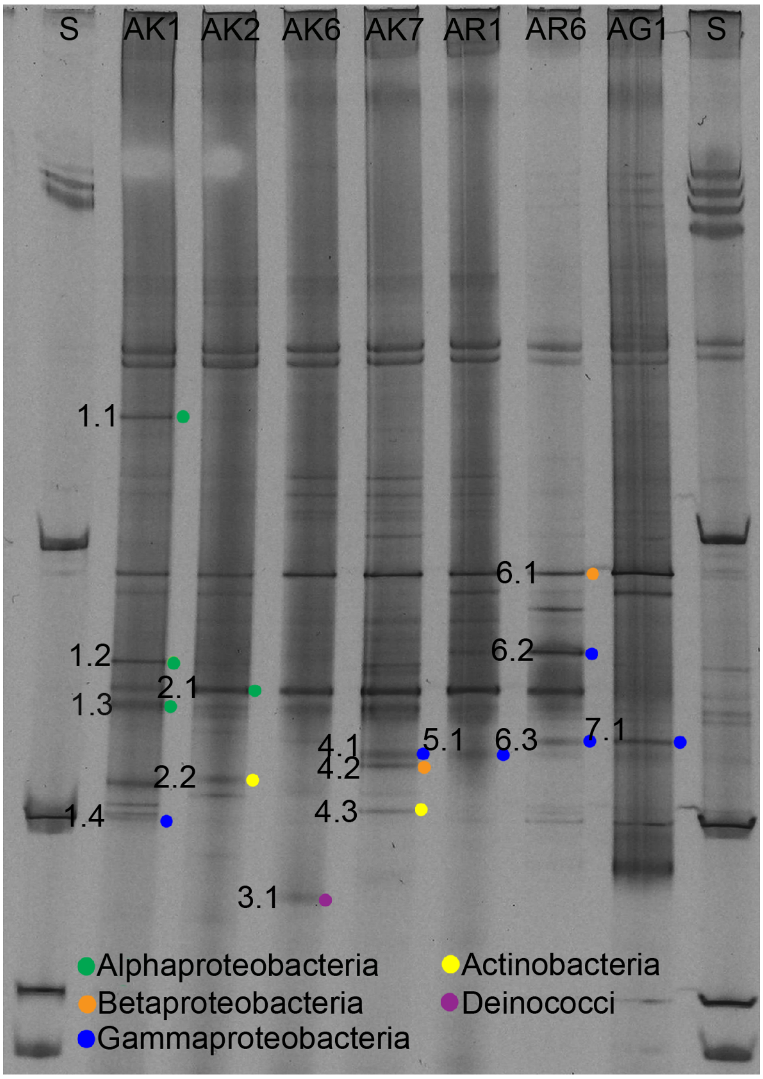

Supplement: S1 Fig — AK1, AK2: exumbrella surface of jellyfish collected in May; AK6, AK7: the exumbrella surface of jellyfish collected in June; AR1: sample of oral arms of jellyfish collected in May; AR6: the oral arms of jellyfish collected in June; AG1: the gastral cavity mucus sample; S: standard. The numbers on the figure represent bands that were cut from the gel and successfully sequenced; color dots place sequence in one of the bacterial groups. (PDF) [file pone.0198056.s009.pdf]
