# Supplementary material for: Bacteria associated with moon jellyfish during bloom and post-bloom periods in the Gulf of Trieste (northern Adriatic)
Source: PLoS One. 2019 Jan 15;14(1):e0198056. doi: 10.1371/journal.pone.0198056 (PMC6333360; doi:10.1371/journal.pone.0198056)
Supplement: S1 Protocol — (PDF) [file pone.0198056.s010.pdf]

S1 Protocol. Detailed description of DNA extraction methods, with modifications.

A

- ***DNA extraction from bacterial isolates: Modified Chelex-based procedure*** (Giraffa et al., 2000).

Washed cells were re-suspended in 200 µl of 5% Chelex solution. The suspension was incubated for 15 min at 99 °C and transferred at 4 °C for 10 min, and was centrifuged at 1500 xg for 10 min. The water phase with dissolved DNA was transferred to a new tube and stored at -20 °C until further downstream applications.

B

- ***Jellyfish-associated bacterial community DNA extraction : Modified protocol for bacterial DNA extraction with CTAB (cetyl- trimethyl-ammonium bromide)*** (Hao, 2014).

Samples were added into a tube containing 2% CTAB solution (1.4 M NaCl, 100 mM Tris_Cl pH= 8, 2% CTAB, 20 mM EDTA pH=8, 0.2% β- mercaptoethanol), and were incubated at 65°C for 1 h. Afterwards, a STE buffer (6.7% sucrose, 50 mM Tris-Cl pH= 8, 1 mM EDTA pH= 8) and proteinase K (100 µg/ml final concentration, Sigma) were added and the mixture was incubated at 55°C overnight. Following chloroform-isoamylacohol (24:1, v/v, Sigma) and phenol- chloroform- isoamylacohol (25:24:1, v/v, Sigma) purification steps, DNA was precipitated at - 20°C overnight with isopropanol. The pellet was washed with 70% ice-cold ethanol and dried in a speed vac. The precipitated DNA was re-suspended in 0.22 µm pre-filtered, autoclaved 1X TE buffer, and kept at – 20°C.
