# Supplementary material for: Bacteria associated with moon jellyfish during bloom and post-bloom periods in the Gulf of Trieste (northern Adriatic)
Source: PLoS One. 2019 Jan 15;14(1):e0198056. doi: 10.1371/journal.pone.0198056 (PMC6333360; doi:10.1371/journal.pone.0198056)
Supplement: S2 Protocol — (PDF) [file pone.0198056.s011.pdf]

S2 Protocol. PCR reaction mix composition and PCR temperature cycling conditions.

A

- ***Amplification of bacterial 16S rRNA genes extracted from bacterial isolates***

Bacterial 16S rRNA genes were amplified using universal bacterial primers 27F and 1492R (Lane, 1991). The PCR reaction mix (50 µl) contained 2 µl (50-100 ng) of extracted genomic DNA, 1x reaction buffer (TrisKCl-$MgCl_2$), 2 mM $MgCl_2$, 0.2 mM dNTP, 1 µM of each primer, and Taq polymerase (5U/µl, Fermentas).

The PCR temperature cycling conditions were as follows: initial denaturation at 94°C for 2 min; 30 cycles of denaturation at 94°C for 1 min, annealing at 55°C for 2 min, and elongation at 72°C for 2 min The final cycle was followed by extension at 72°C for 5 min.

B

- ***Amplification of bacterial 16S rRNA genes of total bacterial community for Denaturing Gradient Gel Electrophoresis***

Standard PCR- DGGE strategy

For DGGE analysis of jellyfish-associated and seawater's total bacterial community, the bacterial 16S rRNA genes were amplified using a universal primer set, 341F with a 40 bp GC-clamp and 907R as described before (Muyzer et al., 1993; Muyzer and Smalla, 1998).

The PCR reaction mix with a final volume 50 µl contained 2 µl of extracted DNA (50–100 ng), 1x reaction buffer (Tris KCl-$MgCl_{2,}$ Fermentas), 1.5 mM $MgCl_2$ (Fermentas), 0.2 mM dNTP (Fermentas), 0.5 µM of each primer (Sigma), 0.38 µg/ml BSA (Fermentas), and Taq polymerase (5 U/µl, Fermentas).

The PCR touchdown protocol according to Don *et al.* (Don et al., 1991) was used: with initial denaturation at 94°C for 5 min, followed by 10 touchdown cycles (1°C decrease of annealing

temperature every cycle) and 20 standard cycles: denaturation for 1 min at 94°C, primer annealing for 1 min at 55°C, and primer extension for 3 min at 72°C. The last cycle was followed by 5 min incubation at primer extension temperature of 72°C.

Nested PCR-DGGE strategy

- First step: Bacterial 16S rRNA genes were first amplified with universal primer set, 27F and 1492R. The PCR reaction mix with a final volume 50 µl contained 2 µl of extracted DNA (50–100 ng), 1x reaction buffer (Tris KCl-$MgCl_2$, Fermentas), 1.5 mM $MgCl_2$ (Fermentas), 0.2 mM dNTP (Fermentas), 0.5 µM of each primer (Sigma), 0.38 µg/ml BSA (Fermentas), and Taq polymerase (5 U/µl, Fermentas).

  The PCR temperature cycling conditions were as follows: initial denaturation for 5 min at 95°C, followed by 25 standard cycles: denaturation at 95°C for 1 min, primer annealing for 1 min at 50°C, and primer extension at 72°C for 2 min. The last cycle was followed by 5 min incubation at the primer extension temperature of 72°C.
- Second step: nested amplification was performed using a DGGE primer set, PCR mixture, and a touchdown annealing protocol, as described above in this section (Standard PCR-DGGE strategy).

C

- ***Amplification of bacterial 16S rRNA genes from excised bands from DGGE gel***

The eluted DNA was re-amplified using primer set 341F and 907R. The PCR reaction mix with a final volume 50 µl contained 2 µl of extracted DNA, 1x reaction buffer (Tris KCl-$MgCl_2$, Fermentas), 1.5 mM $MgCl_2$ (Fermentas), 0.2 mM dNTP (Fermentas), 0.5 µM of each primer (Sigma), 0.38 µg/ml BSA (Fermentas), and Taq polymerase (5 U/µl, Fermentas).

The cycling protocol used was as follows: initial denaturation at 94°C for 2 min; 30 cycles of denaturation at 94°C for 1 min, annealing at 55°C for 2 min, and elongation at 72°C for 2 min. The final cycle was followed by extension at 72°C for 5 min.

D

- ***Amplification of bacterial 16S rRNA gene for clone library construction***

Standard PCR- clone library approach

For clone library construction, bacterial 16S rRNA gens were amplified using the same DNA as for DGGE and universal primer set, 27F and 1492R. The PCR reaction mix with a final volume 50 µl contained 2 µl of extracted DNA, 1x reaction buffer (Tris KCl-$MgCl_2$, Fermentas), 1.5 mM $MgCl_2$ (Fermentas), 0.2 mM dNTP (Fermentas), 0.5 µM of each primer (Sigma), 0.38 µg/ml BSA (Fermentas), and Taq polymerase (5 U/µl, Fermentas).

The cycling protocol used was as follows: initial denaturation at 95°C for 2 min; 30 cycles of denaturation at 95°C for 30 s, annealing at 50°C for 30 s, and elongation at 72°C for 2 min. The final cycle was followed by extension at 72°C for 7 min.

Nested PCR- Clone library approach (Giloteaux et al., 2010) with modifications

First step: bacterial 16S rRNA gene was first amplified with a universal primer set, 27F and 1492R, using same protocol and reaction mix as in the first amplification step of the nested PCR-DGGE strategy.

Second step: nested amplification was performed using primers 341F and 907R. The PCR reaction mixture and cycling protocol were the same as used for standard clone library approach.
